# Susceptibility to *Plasmodium falciparum* Malaria: Influence of Combined Polymorphisms of IgG3 Gm Allotypes and Fc Gamma Receptors IIA, IIIA, and IIIB

**DOI:** 10.3389/fimmu.2020.608016

**Published:** 2020-12-23

**Authors:** Abdou Khadre Dit Jadir Fall, Celia Dechavanne, Audrey Sabbagh, Evelyne Guitard, Jacqueline Milet, André Garcia, Jean-Michel Dugoujon, David Courtin, Florence Migot-Nabias

**Affiliations:** ^1^ Université de Paris, Institut de Recherche pour le Développement (IRD), UMR 261 MERIT, Paris, France; ^2^ CNRS UMR 5288 Laboratoire d’Anthropologie Moléculaire et d’Imagerie de Synthèse (AMIS), Université Paul Sabatier Toulouse III, Toulouse, France

**Keywords:** malaria, IgG polymorphism, Gm allotypes, Fc gamma receptor, Benin, generalized multifactor dimensionality reduction

## Abstract

The binding of immunoglobulin (Ig) to Fc gamma receptors (FcgR) at the immune cell surface is an important step to initiate immunological defense against malaria. However, polymorphisms in receptors and/or constant regions of the IgG heavy chains may modulate this binding. Here, we investigated whether polymorphisms located in FcgR and constant regions of the heavy chain of IgG are associated with susceptibility to *P. falciparum* malaria. For this purpose, a clinical and parasitological follow-up on malaria was conducted among 656 infants in southern Benin. G3m allotypes (from total IgG3) were determined by a serological method of hemagglutination inhibition. FcgRIIA 131R/H and FcgRIIIA 176F/V genotypes were determined using the TaqMan method and FcgRIIIB NA1/NA2 genotypes were assessed by polymerase chain reaction using allele-specific primers. Association analyses between the number of malaria infections during the follow-up and polymorphisms in IgG G3m allotypes and FcgR were studied independently by zero inflated binomial negative regression. The influence of combinations of G3m allotypes and FcgRIIA/FcgRIIIA/FcgRIIIB polymorphisms on the number of *P. falciparum* infections, and their potential interaction with environmental exposure to malaria was assessed by using the generalized multifactor dimensionality reduction (GMDR) method. Results showed that individual carriage of G3m24 single allotype and of G3m5,6,10,11,13,14,24 phenotype was independently associated with a high risk of malaria infection. A risk effect for G3m6 was observed only under high environmental exposure. FcgRIIIA 176VV single genotype and combined carriage of FcgRIIA 131RH/FcgRIIIA 176VV/FcgRIIIB NA1NA2, FcgRIIA 131HH/FcgRIIIA 176FF/FcgRIIIB NA1NA1, FcgRIIA 131HH/FcgRIIIA 176VV/FcgRIIIB NA2NA2 and FcgRIIA 131HH/FcgRIIIA 176VV/FcgRIIIB NA1NA2 genotypes were related to a high number of malaria infections. The risk was accentuated for FcgRIIIA 176VV when considering the influence of environmental exposure to malaria. Finally, the GMDR analysis including environmental exposure showed strengthened associations with a malaria risk when FcgRIIA/FcgRIIIA/FcgRIIIB genotypes were combined to G3m5,6,11,24 and G3m5,6,10,11,13,15,24 phenotypes or G3m10 and G3m13 single allotypes. Our results highlight the relevance of studying IgG heavy chain and FcgR polymorphisms, independently as well as in combination, in relation to the individual susceptibility to *P. falciparum* infection. The intensity of individual exposure to mosquito bites was demonstrated to impact the relationships found.

## Introduction

Malaria remains the most lethal parasitic disease in the world. According to the latest world malaria report in 2019, 228 million estimated cases of malaria occurred in 2018 while the estimated number of deaths remained a concern, 429 000 in 2017 and 405 000 in 2018 (1). Data for the 2015–2018 period highlight that no significant progress in reducing global malaria cases was made. Therefore, it seems urgent to identify new targets and new tools to fight the disease.

The pioneering work of Cohen et al. in 1960 demonstrated that IgG from malaria immune Gambian adults contributed to diminish the *P. falciparum* parasitemia when transferred to non-immune African infected children (2). Since then, there has been an increased interest in exploring the role of IgG in malaria immunity. Namely, the same experimentation was made with IgG from malaria immune African adults passively transferred to Thai patients, demonstrating that efficacy was independent of the type of infecting isolate (3). Further studies established that firstly, cytophilic IgG1 and IgG3 isotypes were mostly associated with *P. falciparum* malaria protection (4–6) including protection of the newborn partly conferred by transplacental transfer of malaria-specific IgG3 (7). Secondly, they showed that it was crucial to investigate the functionality, and not only the levels of IgG directed to asexual stages of *P. falciparum* when evaluating malaria protection parameters (8).

IgG can act on *P. falciparum* in two ways: directly by agglutinating the parasites and therefore preventing their reinvasion of red blood cells and indirectly by binding to Fc gamma receptors (FcgR) expressed at the surface of immune cells such as monocytes, macrophages, or neutrophils (9). This fixation triggers cell activation signals and immune response (opsonization, phagocytosis, reactive oxygen species-ROS, and nitric oxide-NO production). FcgR are important in providing a significant link between the humoral and cellular immunity by bridging the interaction between specific antibodies and effector cells. Nevertheless, genetic variability in constant regions of IgG heavy chains (10) and in FcgR (11) could modulate the susceptibility to malaria infections.

Indeed, most of the interactions between FcgR and IgG involve constant regions (CH1, CH2, and CH3) of the heavy IgG chains. Allelic variations found in the CH1, CH2, and CH3 IgG chains can lead to variations in the amino acid sequences of IgG subclasses and therefore in their efficiency to bind to their receptors. These amino acid changes are responsible for antigenic determinants named human immunoglobulin Gm (gamma markers) allotypes. There are four allotypes for IgG1 in the CH1 and CH3 constant domains called G1m [1, 2, 3, 17], one allotype for IgG2 in the CH2 constant domain called G2m23 and thirteen allotypes for IgG3 in the CH2 and CH3 constant domains called G3m [5, 6, 10, 11, 13, 14, 15, 16, 21, 24, 26, 27, 28] (12, 13). The most polymorphic G3m allotypes are distinguished by variations on nine amino acids (13, 14). Gm allotypes, which are inherited in fixed combinations called haplotypes, vary qualitatively and quantitatively according to human population groups (15).

Facer (16) was the first author to highlight the relationship between Gm allotypes and malaria. She showed, in a sample of Gambian children with past or acute *P. falciparum* malaria, a preferential expression of G3m10, G3m11, and G3m14 allotypes associated with a risk of anemia (16). According to Pandey et al. (17), G3m6 carrying haplotypes may explain the difference in susceptibility to malaria infection between the Fulani and Masaleit ethnic groups in Sudan. Indeed, a lower frequency of G3m6 in Fulani was associated with a lower parasitamia compared to Masaleit. Migot-Nabias et al. (10) demonstrated the existence of an inverse relationship between the carriage of the Gm5,6,13,14; 1,17 phenotype (G3m; G1m) and the presence of uncomplicated malaria in Benin, while for Giha et al. (18) the Gm5,6,13,14; 1,17 phenotype was associated with a higher incidence of malaria in Sudan. Finally, Pandey et al. (19) showed that the carriage of Gm5,13,14; 3; 23 phenotype (G3m; G1m; G2m) was associated with a high level of IgG1 to *P. vivax Pv*MSP1-19 and *Pv*AMA-1 antigens, response considered as protective against malaria.

The FcgRs are key components of the immune response, by operating on activation and modulation of the pro-inflammatory and cytotoxic pathways, by influencing the number of white blood cells as well as the transport of circulating antibodies (20, 21). In the family of FcgR receptors, FcgRIIA, FcgRIIIA, and FcgRIIIB appear to play an important role in malaria susceptibility.

FcgRIIA initiates endocytosis, phagocytosis, and the release of inflammatory mediators. There are two variants for FcgRIIA, 131R (Arginine) and 131H (Histidine) firstly described according to their IgG2 binding efficiency, which is more affine for the 131H variant (22). Binding affinity for IgG is more important for IgG3 and IgG1 than for IgG4 and IgG2 (23). Ouma et al. (24) showed that 131R homozygosity confers protection against high parasite densities in contrast to 131H homozygosity.

FcgRIIIA receptor plays an important role in phagocytosis and degranulation and presents two allotypes variants consisting in either F (Phenylalanine) or V (Valine) in position 176, the 176V variant offering a better affinity for IgG1 (25).

FcgRIIIB is a C-terminus linked glycosylphosphatidylinositol (GPI) receptor expressed at the surface of neutrophils and eosinophils. A strong affinity for IgG1 and IgG3 was described (11). The FcgRIIIB presents a polymorphism named NA1/NA2 that refers to neutrophil antigen (NA) 1 and 2 forms which differ in amino acids at positions 65 and 82 in two extra-glycosylation sites (11, 26, 27). The FcgRIIIB NA1 form is capable of better ingestion of IgG1 or IgG3 opsonized particles than the NA2 form (28). The FcgRIIA and FcgRIIIB receptors play an important role in phagocytosis and degranulation. Indeed, in order of decreasing affinity, we have for FcgRIIA, IgG3 > IgG1 > IgG2 = IgG4, for FcgRIIIA, IgG3 > IgG1 >> IgG4 > IgG2 while FcgRIIIB binds generally IgG1 and IgG3 and not IgG2 and IgG4 (23).

Various studies have investigated the relationships between polymorphisms in FcgRIIA, FcgRIIIA, and FcgRIIIB receptors and malaria immunity. Omi et al. (29) showed an association between the FcgRIIIB NA2 allele and an increased susceptibility to cerebral malaria in Thailand. Those results were strengthened by the study of Adu et al. (30) which described an association between FcgRIIIB NA2 allele and a higher risk of malaria infection. Moreover, the authors showed that the FcgRIIA 131R allele was associated with protection against malaria. The presence of the FcgRIIA 131RH heterozygous polymorphism was associated with malaria protection in some studies (31, 32) while for Maiga et al. (33), a marginal protective effect on parasitemia was observed in a Fulani group from Mali harboring the 131RR genotype. Munde et al. (11) demonstrated that the FcgRIIA 131R-FcgRIIIA 176F-FcgRIIIB NA2 haplotype was important in conditioning susceptibility to malaria anemia (or increased levels of circulating parasites) in West Kenya. Ouma et al. (34) reinforced the results obtained from independent analysis of FcgR (31, 32) by showing a negative association between the FcgRIIA 131H-FcgRIIIB NA1 haplotype and severe malaria anemia and a positive one when considering the FcgRIIA 131H-FcgRIIIB NA2 haplotype.

As presented above, the individual susceptibility to malaria has yet been related to polymorphisms located either in the constant domains of the IgG heavy chain or in several FcgR, considered independently. We can hypothesize that the anchoring of IgG on its cellular receptors can be favored according to the respective variants on both sides. For this purpose, the present study investigated the combination of polymorphisms at both IgG constant domains and FcgR with the aim to evaluate their joint impact on susceptibility to *P. falciparum* malaria. More precisely, the relationships between FcgRIIA 131R/H, FcgRIIIA 176 F/V, FcgRIIIB NA1/NA2, Gm allotypes, and malaria phenotypes were determined in a cohort of Beninese infants.

## Methods

### Study Area and Design

The study was conducted in three health centers from the district of Tori Bossito located in southwest Benin, where 656 infants were included at birth (35) and 567 of them followed up from birth to 18 months of age (36). Health workers performed an active follow-up of these infants. It consisted of scheduled home visits every week to detect fever. In case of axillary temperature higher than 37.5°C, a questionnaire was fulfilled and both a *Plasmodium* rapid diagnostic test (RDT) and a thick blood smear examination (TBS) by optical microscopy were performed.

A symptomatic malaria infection was defined by the combined presence of an axillary temperature >37.5°C and a positive RDT and/or a positive TBS. In this case, an antimalarial treatment was administered according to the national guidelines that were applied at the time of the study. It consisted in an artemisinin-based combination therapy (artemether and lumefantrine). TBS were also performed every month to detect asymptomatic malaria infections. In addition, mothers were invited to bring their infants to the health center, at any time, in case of fever (suspected by the mother) or clinical signs, whether or not they were related to malaria, and the same protocol was applied.

Venous blood samples were collected quarterly for hematological and immunological measurements. Venous blood was centrifuged for plasma isolation, and genomic DNA extraction was performed from buffy coat using the QIAamp^®^ DNA blood Mini Kit (QIAGEN) according to the manufacturer’s instructions.

To assess the environmental risk of malaria exposure, environmental (information on house characteristics and its immediate surrounding) and geographical data (satellite images, soil type, watercourse nearby, vegetation index, rainfall) were recorded. Throughout the study, every 6 weeks, human landing catches were performed in several points of the villages to evaluate spatial and temporal variations of *Anopheles* density. Altogether, these data allowed modeling, for each child included in the follow-up, an individual risk of exposure to *Anopheles* bites by means of a space- and time-dependent variable (37).

### IgG Gm Allotypes

The Gm allotype determination was performed for 501 infants (all singletons) for whom a sufficient quantity of plasma (200 µl) was available at 15 months of age. Indeed, plasma samples collected earlier in the follow-up (3, 6, 9, and 12 months) were not suitable for this determination due 1) to a possible presence of IgG from maternal origin bearing their own Gm allotypes and 2) to an insufficient quantity of infant neo-synthesized IgG.

In order to guarantee a highly reliable result, Gm allotype determinations for infants were confirmed by means of their consistency (inheritance) with those of both biological parents. Thereby, Gm determinations of 71 infants were discarded due to the lack of either the paternal (n = 62) or the maternal sample (n = 9).

From the 430 residual samples for whom trios of Gm determinations could be constituted, inconsistent determinations within the father-mother-child trio were observed for 23 cases (5.3%), leading to Gm data for 407 infants that were retained for further analysis.

G1m [1, 2, 3, 17] and G3m [5, 6, 10, 11, 13, 14, 15, 16, 21, 24, 28] allotypes were determined in plasma samples by a standard hemagglutination inhibition method (38). All infants carried the G1m1,17 allele. Considering G3m allotypes, no child presented G3m16 or G3m21, both on the CH2 domain, and absent in populations of sub-Saharan Africa (13). Similarly, due to the uncertain location of the Gm28 allotype on either IgG1 or IgG3 sub-classes among sub-Saharan Africans (13), this allotype was discarded from the analysis. Therefore, the differentiation of infants was based on the remaining G3m allotypes combined into four G3m alleles mostly encountered in Africa, that are G3m5,10,11,13,14, G3m5,6,11,24, G3m5,6,10,11,14, and G3m10,11,13,15 (13, 15). The homozygous or heterozygous carriage of these alleles led to the ten following possible G3m phenotypes: G3m5,10,11,13,14; G3m5,6,10,11,14; G3m5,6,10,11,13,14; G3m5,6,11,24; G3m5,6,10,11,13,14,24; G3m5,6,10,11,14,24; G3m10,11,13,15; G3m5,10,11,13,14,15; G3m5,6,10,11,13,14,15; and G3m5,6,10,11,13,15,24.

### FcgRIIA, FcgRIIIA, and FcgRIIIB Genotyping

Genomic DNA was available for 369 out of the 407 infants for whom Gm data were retained. Among these 369 infants, 368 Gm data were available, while all were genotyped for FcgRIIA, 365 for FcgRIIIA and 353 for FcgRIIIB. However, multivariate models using the four complete FcgR polymorphisms and G3m phenotypes determinations were done on 350 infants.

#### FcgRIIA and FcgRIIIA Genotyping

Single nucleotide polymorphisms (SNP) corresponding to FcgRIIA rs1801274 and FcgRIIIA rs396991 were genotyped by the Applied Biosystems TaqMan SNP Genotyping Assay using predesigned primer/probe sets (C_9077561_20 and C_25815666_10). PCR was performed using the 7900HT Real-time PCR System (Applied Biosystems) according to the following conditions: one cycle at 92°C for 10 min, 40 cycles at 92°C for 15 s, and 60°C for 60 s. The results were then analyzed using the SDS software.

#### FcgRIIIB Genotyping

Two different PCR were performed for the detection of FcgRIIIB NA1 and NA2 alleles, using allele-specific oligonucleotides described previously by Hans and Mehta (39). Regarding NA1, the 5’-CAG TGG TTT CAC AAT GTG AA-3’ (forward) and 5’ CAT GGA CTT CTA GCT GCA CCG 3’ (reverse) primers were used to amplify a DNA fragment of 142 pb. Genomic DNA (100 ng) was added to the reaction mixture containing 100 µM MgCl_2_, 1X buffer solution, 16 μM dNTPs, 2.8 µM sense and antisense primers and 0.5 unit of Taq polymerase. PCR reaction conditions included 1 cycle at 95°C for 5min, followed by 30 cycles at 95°C for 30 s, 55°C for 30 s, and 72°C for 45 s. For NA2, the 5’-CTTC AAT GGT ACA GCG TGC TT-3’ (forward) and 5’-CTG TAC TCT CCA CTG TCG TT-3’ (reverse) primers were used to amplify a DNA sequence of 169 pb. Genomic DNA (100 ng) was added to the reaction mixture containing 45 µM MgCl_2_, 1X buffer solution, 16 μM dNTPs, 1.39 µM sense and antisense primers, and 0.5 unit of Taq polymerase. PCR reaction conditions included one cycle at 95°C for 5min, followed by 30 cycles at 95°C for 30 s, 60°C for 30 s, and 72°C for 45 s. The products of 142 pb and 169 pb were revealed on a 2% agarose gel.

### Statistical Analysis

The clinical phenotype of interest was the number of *P. falciparum* infections per individual during the follow-up. No discrimination was made between symptomatic and asymptomatic infections since the total of symptomatic infections was greater than that of asymptomatic infections (597 *versus* 200 out of 797 infections) and there were more infants presenting only symptomatic infections (n = 153) than infants presenting only asymptomatic infections (n = 14).

The Chi-square test was used to examine differences between proportions and the Mann-Whitney *U*-test was used for comparisons of demographic and clinical characteristics between groups. The genotypic frequencies of FcgRIIA 131R/H, FcgRIIIA 176F/V and FcgRIIIB NA1/NA2 were tested for Hardy-Weinberg equilibrium (HWE).

First, association analyses between the number of malaria infections during the 18-month follow-up and polymorphisms in FcgR and IgG G3m allotypes were studied by zero-inflated binomial negative regression with adjustment on covariates (age of mothers in years and of infants in months, number of antenatal visits, bednet use, birthweight, and environmental exposure). Environmental exposure was categorized into low *versus* high exposure, taking the median as the threshold value. Univariate analyzes on each of the covariates were made and only those with a P value < 0.20 in the univariate model were included in the final multivariate model.

The statistical analysis was carried out using the Stata software version 13.

Finally, to evaluate the influence of combinations of G3m allotypes, FcgRIIA, FcgRIIIA, and FcgRIIIB polymorphisms on the number of *P. falciparum* infections during the follow-up, and their potential interaction with mosquito exposure, we performed multivariate analyses by using the generalized multifactor dimensionality reduction (GMDR) method (40). This non-parametric and genetic model-free approach overcomes some of the limitations of the traditional statistical methods (i.e. sample size limitation) to detect and characterize gene-gene and gene-environment interactions. GMDR is very similar to the original MDR method. However, instead of using the counts of individuals, the GMDR method uses a residual-based score in order to classify the individuals, thus allowing adjustment for discrete and continuous covariates.

Since the GMDR software requires a complete dataset with no missing values for analysis, we removed individuals with missing genotype data for at least two polymorphisms and those with unknown number of antenatal visits (n = 21). The remaining missing data for FcgR polymorphisms were imputed using the R package MICE (Multivariate Imputation by Chained Equations) (41), a multivariate imputation approach that takes into consideration patterns in the data such as linkage disequilibrium.

We performed an exhaustive search of all possible genotype combinations of one to four polymorphisms among those studied (G3m allotypes, FcgRIIA 131R/H, FcgRIIIA 176F/V, and FcgRIIIB NA1/NA2), and model selection and evaluation was carried out using ten-fold cross-validation. Briefly, a GMDR model was developed using 9/10th of the data and a classification error was estimated from this training set. Then, cross-validation methods were used to estimate the prediction error of the selected GMDR model using 1/10th of the data as evaluation data. This procedure was repeated for each of the ten pieces of the data and the classification and prediction errors were averaged across all ten runs. Two parameters were used to evaluate the best models: (a) the testing balanced accuracy (TBA), which is a measure of the degree of accuracy to which the selected interaction correctly predicts the number of malaria infections in the testing sets (value averaged across all ten sets), with 1.00 indicating perfect prediction, and (b) the cross-validation consistency (CVC), which indicates how many times a set of loci is identified across the cross-validation subsets.

Besides TBA and CVC statistics, GMDR also provides a measure of the significance of the identified model, the signal test, a robust nonparametric test implemented in this extension to MDR (40). The model with the highest TBA, the maximum CVC score, and 0.05 or lower P value derived from the sign test was considered as the best model.

In the first round of analysis, the environmental variable quantifying mosquito exposure at individual level was included as a covariate in the GMDR models as well as the following variables: mother and child ages, bednet use, and number of antenatal visits. In the second round of analysis, this environmental variable was tested for its interaction with the gene polymorphisms to detect gene-environment interactions (only four covariates in the GMDR models).

A linear regression analysis was performed for the significant models found in the GMDR analysis to estimate the effect of the combinations on malaria infections.

### Ethics

The University of Abomey-Calavi’s institutional review board and the IRD’s Consultative Ethics Committee approved the study protocol. All women in this study signed an informed consent before enrollment (which also included their infants) with the possibility to withdraw at any time.

## Results

### Participants Characteristics

As presented in **Table 1**, the study was conducted in a sample of Beninese infants presenting malaria infections (n = 260, 70%) or not (n = 109, 30%). The number of infants who had symptomatic and asymptomatic infections during the follow-up was respectively 245 and 106. A majority of infants presenting malaria infections had less than four infections (n = 201, 77.3%), 51 infants (19.6%) had between five and eight infections and eight infants (3.1%) had more than eight infections during the follow-up.

**Table 1:** Characteristics of the 369 study participants.

Characteristics of mothers and infants	*P. falciparum* infections in infants		P-value
	No(n = 109)	Yes(n = 260)	
**Mothers**			
Mother age (mean ± SD in years)	30 (6.07)	26 (5.20)	**0.001** ^a^
Number of antenatal visits (mean ± SD)	4.20 (2.22)	3.43 (1.58)	0.443^a^
Placental malaria (n, %)			0.189^b^
No	99 (27.0)	224 (61.2)	
Yes	9 (2.5)	34 (9.3)	
Maternal education (n, %)			0.781^b^
No education	94 (25.5)	221 (59.9)	
Partial primary	11 (3.0)	25 (6.8)	
Complete primary or more	4 (1.0)	14 (3.8)	
Primigravidae (n, %)			0.078^b^
No	98 (28.5)	215 (58.3)	
Yes	11 (3.0)	45 (12.2)	
**Infants**			
Birth weight (mean ± SD in g)	3049 (418)	2976 (392)	0.589^a^
Infant age (mean ± SD in months)	12 (3.54)	14 (3.06)	<**0.001** ^a^
Sex (n, %):			
Male	55 (50.4)	129 (49.6)	0.882^b^
Female	54 (49.5)	131 (50.4)	
Ethnic group (n, %)^c^:			
Tori	77 (70.6)	195 (51.9)	0.156^b^
Fon	9 (8.2)	27 (10.4)	
Others	23 (21.1)	34 (13.1)	
Bednet use (mean of use ± SD)	0.77 (0.41)	0.71 (0.45)	**0.023** ^a^
Mosquito exposure (mean of exposure ± SD)	0.42 (0.50)	0.53 (0.50)	**0.048** ^a^
***P. falciparum* infections in infants (n, %)**			
1 to 4 infections	_	201 (77.3)	
5 to 8 infections		51(19.6)	
9 to 16 infections		8 (3.1)	

a Statistical significance determined using Mann-Whitney U-test.

b Statistical significance determined by χ2 analysis.

c 4 missing values.

In bold: significant P value at the 0.05 threshold.

Infants belonged mostly to the Tori ethnic group and no difference appeared between ethnic groups regarding malaria infection (P = 0.156). There were almost as many girls as boys and infants distributed equally in *P. falciparum* infected and non-infected groups (P = 0.842).

Interestingly, infants from the non-infected group were younger (P < 0.001), had more bednet use (P = 0.023), and were less exposed to mosquitoes (P = 0.048) and their mothers were older (P = 0.001) than infants from the *P. falciparum* infected group.

### G3m Allotypes and Malaria Infection

#### Distribution of G3m Phenotypes in the Study Group

**Figure 1:**
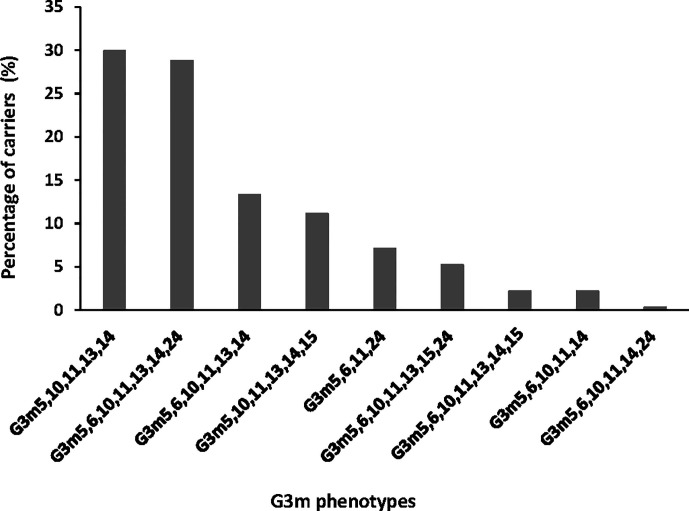
Distribution of G3m phenotypes in the study group.

Nine G3m phenotypes were observed in the study population, among which six presented a frequency above 5% and were G3m5,10,11,13,14 (n = 110, 29.9%), G3m5,6,10,11,13,14,24 (n = 106, 28.8%), G3m5,6,10,11,13,14 (n = 49, 13.3%), G3m5,10,11,13,14,15 (n = 41, 11.1%), G3m5,6,11,24 (n = 26, 7.1%), and G3m5,6,10,11,13,15,24 (n = 19, 5.2%) (**Figure 1**). The three remaining combinations were G3m5,6,10,11,13,14,15 (n = 8, 2.2%), G3m5,6,10,11,14 (n = 8, 2.2%), and G3m5,6,10,11,14,24 (n = 1, 0.3%).

#### G3m Allotypes and Malaria Infections

Carriage of G3m allotypes both in single form and in combinations was analyzed through a zero-inflated binomial regression model in order to explain the number of malaria infections per infant during the follow-up. We introduced a term of interaction between the genetic and the environmental variables in the model in order to explore the potential effect of G3m allotypes varying according to the intensity of individual exposure to mosquito bites.

##### Association Between G3m Allotypes and Malaria Infections

The six most prevalent G3m phenotypes (frequency above 5%) were analyzed.

Only G3m5,6,10,11,13,14,24 was associated with a greater risk of malaria infections (n = 106, IRR = 1.295, 95% CI = 1.093;1.535, P = 0.003, **Table 2**).

**Table 2:** Association between G3m allotypes and malaria infections.

G3m allotypes	n	IRR	CI 95%	P value
G3m phenotypes with frequency at least 5%				
G3m5,10,11,13,14	110	0.952	0.793; 1.149	0.626
G3m5,6,10,11,13,14,24	**106**	**1.295**	**1.093; 1.535**	**0.003**
G3m5,6,10,11,13,14	49	0.821	0.646; 1.043	0.100
G3m5,10,11,13,14,15	41	0.252	0.653; 1.171	0.370
G3m5,6,11,24	26	0.938	0.674; 1.302	0.708
G3m5,6,10,11,13,15,24	19	0.853	0.577; 1.259	0.424
G3m single allotypes				
G3m6	217	1.089	0.918; 1.292	0.323
G3m10	342	1.065	0.765; 1.482	0.708
G3m13	333	1.016	0.777; 1.330	0.903
G3m14	323	1.109	0.858; 1.433	0.426
G3m15	68	0.855	0.684; 1.069	0.171
G3m24	**152**	**1.195**	**1.016; 1.406**	**0.031**

This table presents the zero inflated negative binomial model obtained through the control variables mother age (in years), child age (in months), bednet use, environmental exposure, and number of antenatal visits. The reference groups were the absence of the respective models for each G3m phenotype or single allotype. G3m5 and G3m11 were not analyzed individually because the whole population group carries them.

In bold: significant P value at the 0.05 threshold.

The analysis of the carriage of single G3m6, G3m10, G3m13, G3m14, G3m15 and G3m24 allotypes showed that G3m24 is associated with an increased risk of malaria infections (n = 152, IRR = 1.195, 95% CI = 1.016;1.406, P = 0.031, **Table 2**).

To explore the potential role of environmental exposure on this effect, we introduced an interaction term in the analyses.

##### Interaction Between G3m Allotypes—Environmental Exposure and Malaria Infections

**Table 3** confirmed that under conditions of high level of environmental exposure to mosquito bites, G3m5,6,10,11,13,14,24 is associated with an increased risk of malaria infection (n = 56, IRR = 1.648, 95% CI = 1.312;2.070, P < 0.001) as well as the carriage of G3m6 (n = 116, IRR = 1.350, 95% CI = 1.059;1.720, P = 0.015) and of G3m24 (n = 78, IRR = 1.479, 95% CI = 1.183;1.850, P = 0.001).

**Table 3:** Interaction between G3m allotypes—environmental exposure and malaria infections.

G3m allotypes*exposure to malaria	n	IRR	95% CI	P value
G3m phenotypes with frequency at least 5%				
Ref. No G3m5,10,11,13,14*low exposure				
G3m5,10,11,13,14*low exposure	61	0.893	0.677; 1.179	0.427
G3m5,10,11,13,14*high exposure	49	1.209	0.927; 1.577	0.160
Ref. No G3m5,6,10,11,13,14,24*low exposure				
G3m5,6,10,11,13,14,24*low exposure	50	1.056	0.803; 1.389	0.693
G3m5,6,10,11,13,14,24*high exposure	**56**	**1.648**	**1.312; 2.070**	**<0.0001**
Ref. No G3m5,6,10,11,13,14*low exposure				
G3m5,6,10,11,13,14*low exposure	19	1.097	0.750; 1.605	0.632
G3m5,6,10,11,13,14*high exposure	30	0.932	0.685; 1.269	0.659
Ref. No G3m5,10,11,13,14,15*low exposure				
G3m5,10,11,13,14,15*low exposure	21	1.042	0.658; 1.651	0.859
G3m5,10,11,13,14,15*high exposure	20	1.018	0.695; 1.491	0.925
Ref. No G3m5,6,11,24*low exposure				
G3m5,6,11,24*low exposure	12	1.095	0.656; 1.828	0.728
G3m5,6,11,24*high exposure	14	1.059	0.682; 1.644	0.797
Ref. No G3m5,6,10,11,13,15,24*low exposure				
G3m5,6,10,11,13,15,24*low exposure	11	0.784	0.420; 1.466	0.448
G3m5,6,10,11,13,15,24*high exposure	8	1.119	0.674; 1.859	0.663
G3m single allotypes				
Ref. No G3m6*low exposure				
G3m6*low exposure	101	1.087	0.837; 1.411	0.529
G3m6*high exposure	**116**	**1.350**	**1.059; 1.720**	**0.015**
Ref. No G3m10*low exposure				
G3m10*low exposure	171	0.895	0.537; 1.491	0.671
G3m10*high exposure	171	1.137	0.685; 1.886	0.618
Ref. No G3m13*low exposure				
G3m13*low exposure	167	0.807	0.540; 1.206	0.297
G3m13*high exposure	166	1.048	0.706; 1.555	0.814
Ref. No G3m14*low exposure				
G3m14*low exposure	160	1.056	0.703; 1.585	0.792
G3m14*high exposure	163	1.336	0.895; 1.993	0.156
Ref. No G3m15*low exposure				
G3m15*low exposure	37	0.897	0.623; 1.241	0.465
G3m15*high exposure	31	1.053	0.775; 1.432	0.737
Ref. No G3m24*low exposure				
G3m24*low exposure	74	1.020	0.789; 1.317	0.878
G3m24*high exposure	**78**	**1.479**	**1.183; 1.850**	**0.001**

This table presents the zero inflated negative binomial model obtained through the control variables mother age (in years), child age (in months), number of antenatal visits, and the use of bednet. The interaction between G3m allotypes and the environmental exposure was studied. The reference groups were indicated in each case.

In bold: significant P value at the 0.05 threshold.

### FcgRIIA 131R/H, FcgRIIIA 176 F/V, and FcgRIIIB NA1/NA2 Polymorphisms

#### Distribution of FcgR Polymorphisms in the Study Group

The observed distribution of FcgRIIA 131R/H and FcgRIIIA 176F/V genotypes in the whole study group showed consistency with HWE (both P > 0.10). The FcgRIIIB NA1/NA2 genotype distribution revealed significant deviation from HWE expectations which was also observed by Munde et al. (11) (P < 0.001, **Table 4**).

**Table 4:** Distribution of FcgR genotypes in the study group.

	FcgRIIA 131 R/H			FcgRIIIA 176 F/V			FcgRIIIB NA1/NA2		
Variations	rs1801274			rs396991					
Genotypes	HH	RH	RR	FF	FV	VV	NA1NA1	NA1NA2	NA2NA2
Number of infants (%)	80 (21.7%)	194 (52.6%)	95 (25.7%)	165 (45.2%)	165 (45.2%)	35 (9.6%)	106 (30.0%)	131 (37.1%)	116 (32.9%)
Minor allele frequency (minor allele)		0.48 (H)			0.32 (V)			0.48 (NA1)	
HWE test ^a^ χ^2^ (P-value, df = 2)	χ^2^ = 1.04 (P > 0.10)			χ^2^ = 0.46 (P > 0.10)			χ^2^ = 23.35 (P < 0.001)		

a Hardy-Weinberg equilibrium test.

#### FcgR Polymorphisms and Malaria Infections

Association between FcgRIIA 131R/H, FcgRIIIA 176F/V, and FcgRIIIB NA1/NA2 polymorphisms and the number of malaria infections was determined using zero-inflated binomial regression models (with and without the interaction between the genetic and environmental variables).

##### Association Between FcgR Polymorphisms and Malaria Infections

We determined if individual FcgR polymorphisms were associated with malaria infections (**Table 5**). None of the FcgRIIA 131RH, 131RR, nor 131HH genotypes was associated with the number of malaria infections. However, infants carrying FcgRIIIA 176VV compared to FcgRIIIA 176FF had a higher risk of infection (n = 35, IRR = 1.344, 95% CI = 0.303;3.308, P = 0.035) while the FcgRIIIB NA1NA2 was associated with a lower number of infections compared to NA2NA2 (n = 131, IRR = 0.812, 95% CI = 0.670;0.985, P = 0.035).

**Table 5:** Association between each FcgRIIA, FcgRIIIA, and FcgRIIIB genotype and malaria infections.

FcgR polymorphisms	n	IRR	95% CI	P value
FcgRIIA 131 R/H model				
131RR (reference)	95			
131RH	194	1.168	0.955; 1.429	0.129
131HH	80	8.435	0.929; 1.523	0.929
FcgRIIIA 176 F/V model				
176FF (reference)	165			
176FV	165	0.948	0.200; 1.069	0.071
176VV	**35**	**1.344**	**0.303; 3.308**	**0.035**
FcgRIIIB NA1/NA2 model				
NA2NA2 (reference)	116			
NA1NA2	**131**	**0.812**	**0.670; 0.985**	**0.035**
NA1NA1	106	0.903	0.735; 1.109	0.332

This table presents the zero inflated negative binomial individual models obtained through the control variables mother age (in years), child age (in months), number of antenatal visits, environmental exposure, and bednet use. The references are indicated for FcgRIIA R/H, FcgRIIIA F/V, and FcgRIIIB NA1/NA2.

In bold: significant P value at the 0.05 threshold.

**Figure 2:**
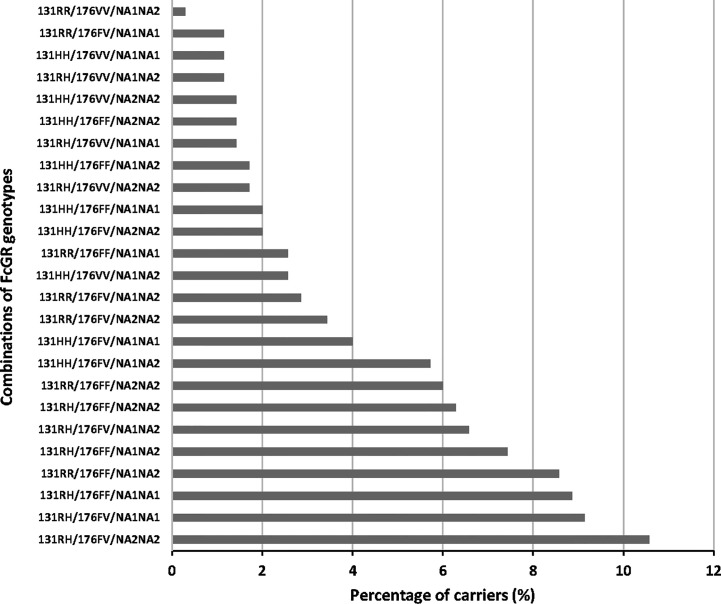
Distribution of FcgRIIA/FcgRIIIA/FcgRIIIB phenotypes in the study group.

Finally, we analyzed the influence of FcgRIIA/RIIIA/RIIIB genotype combinations on the occurrence of malaria infections. The distribution of these combinations is shown in **Figure 2**, where 25 out of the 27 possible combinations are present in the study group, the three most prevalent of them being 131RH/176FV/NA2NA2 (10.6%), 131RH/176FV/NA1NA1 (9.1%), and 131RH/176FF/NA1NA1 (8.9%). No individual presented the 131RR/176VV/NA1NA1 and 131RR/176VV/NA2NA2 genotype combinations. Compared to the reference 131RR/176FF/NA2NA2, a higher risk of malaria infection was found for carriers of the following genotype combinations: 131RH/176VV/NA1NA2 (n = 4, IRR = 2.035, 95% CI = 1.126;3.677, P = 0.019), 131HH/176VV/NA1NA2 (n = 9, IRR = 1.842, 95% CI = 1.016;3.339, P = 0.044), 131HH/176VV/NA2NA2 (n = 5, IRR = 1.933, 95% CI =1.087;3.437, P = 0.025) and 131HH/176FF/NA1NA1 (n = 7, IRR = 2.110, 95% CI = 1.039;4.283, P = 0.039) (**Table 6**). To explore the potential role of environmental exposure on this effect, we introduced an interaction term in the analyses.

**Table 6:** Association between FcgRIIA/FcgRIIIA/FcgRIIIB combined genotypes and malaria infections.

FcgR polymorphisms combination models	n	IRR	95% CI	P value
131RR/176FF/NA2NA2 (reference)	21			
131RR/176FF/NA1NA2	30	1.091	0.684; 1.739	0.713
131RR/176FF/NA1NA1	9	1.456	0.842; 2.520	0.179
131RR/176FV/NA2NA2	12	1.426	0.819; 2.481	0.209
131RR/176FV/NA1NA2	10	0.495	0.214; 1.148	0.102
131RR/176FV/NA1NA1	4	0.980	0.397; 2.415	0.965
131RR/176VV/NA1NA2	1	1.053	0.306; 3.616	0.934
131RH/176FF/NA2NA2	22	1.459	0.934; 2.279	0.096
131RH/176FF/NA1NA2	26	1.036	0.655; 1.639	0.878
131RH/176FF/NA1NA1	31	1.331	0.854; 2.075	0.206
131RH/176FV/NA2NA2	37	1.365	0.879; 2.119	0.165
131RH/176FV/NA1NA2	23	1.014	0.624; 1.649	0.953
131RH/176FV/NA1NA1	32	1.186	0.748; 1.881	0.467
131RH/176VV/NA2NA2	6	0.866	0.295; 2.539	0.795
131RH/176VV/NA1NA2	**4**	**2.035**	**1.126; 3.677**	**0.019**
131RH/176VV/NA1NA1	5	0.684	0.203; 2.307	0.541
131HH/176FF/NA2NA2	5	1.919	0.922; 3.995	0.081
131HH/176FF/NA1NA2	6	0.628	0.214; 1.839	0.397
131HH/176FF/NA1NA1	**7**	**2.110**	**1.039; 4.283**	**0.039**
131HH/176FV/NA2NA2	7	1.064	0.453; 2.496	0.886
131HH/176FV/NA1NA2	20	1.180	0.727; 1.917	0.501
131HH/176FV/NA1NA1	14	0.780	0.436; 1.395	0.403
131HH/176VV/NA2NA2	**5**	**1.933**	**1.087; 3.437**	**0.025**
131HH/176VV/NA1NA2	**9**	**1.842**	**1.016; 3.339**	**0.044**
131HH/176VV/NA1NA1	4	1.838	0.877; 3.851	0.106

This table presents the zero inflated negative binomial model obtained through the control variables age (mother and child), antenatal visits, mosquito exposure, and mosquito net possession for the FcgRIIA/FcgRIIIA/FcgRIIIB genotype models. The reference was the absence of HH/FF/NA2NA2.

In bold: significant P value at the 0.05 threshold.

##### Interaction Between FcgR Polymorphisms—Environmental Exposure and Malaria Infections

Results confirmed the higher risk of infection associated with FcgRIIIA 176VV compared to 176FF when exposed to high levels of mosquito bites (n = 17, IRR = 1.722, 95% CI = 1.213;2.445, P = 0.002). A similar pattern, barely significant, was observed for infants carrying FcgRIIIB NA2NA2 compared to FcgRIIIB NA1NA1 (n = 51, IRR = 1.372, 95% CI = 0.998;1.885, P = 0.051) (**Table 7**).

**Table 7:** Interaction between FcgRIIA, FcgRIIIA, and FcgRIIIB genotypes—environmental exposure and malaria infections.

FcgR genotypes*exposure to malaria	n	IRR	95% CI	P value
FcgRIIA model				
FcgRIIA 131RR*low exposure (reference)	46			
FcgRIIA 131RR*high exposure	49	0.824	0.587; 1.155	0.263
FcgRIIA 131RH*low exposure	104	0.885	0.668; 1.173	0.399
FcgRIIA 131RH*high exposure	90	1.266	0.970; 1.652	0.081
FcgRIIA 131HH*low exposure	34	0.972	0.656; 1.440	0.890
FcgRIIA 131HH*high exposure	46	1.213	0.892; 1.650	0.218
FcgRIIIA model				
FcgRIIIA 176FF*low exposure (reference)	76			
FcgRIIIA 176FF*high exposure	89	1.072	0.847; 1.357	0.559
FcgRIIIA 176FV*low exposure	78	0.833	0.640; 1.083	0.174
FcgRIIIA 176FV*high exposure	87	1.124	0.884; 1.430	0.337
FcgRIIIA 176VV*low exposure	18	1.022	0.646; 1.616	0.924
FcgRIIIA 176VV*high exposure	**17**	**1.722**	**1.213; 2.445**	**0.002**
FcgRIIIB model				
FcgRIIIB NA2NA2*low exposure (reference)	65			
FcgRIIIB NA2NA2*high exposure	51	1.227	0.932; 1.617	0.144
FcgRIIIB NA1NA2*low exposure	66	0.814	0.610; 1.085	0.161
FcgRIIIB NA1NA2*high exposure	65	0.997	0.764; 1.299	0.983
FcgRIIIB NA1NA1*low exposure	48	0.894	0.649; 1.233	0.497
FcgRIIIB NA1NA1*high exposure	58	1.115	0.847; 1.468	0.436
FcgRIIIB NA1NA1*low exposure (reference)	48			
FcgRIIIB NA2NA2*high exposure	51	1.372	0.998; 1.885	0.051

This table presents the zero inflated negative binomial model obtained through the control variables mother age (in years), child age (in months), number of antenatal visits mosquito, and bednet use. The interaction between FcgR polymorphisms and environmental exposure was studied. The reference was the absence of estimations in FcgRIIA R/H, FcgRIIIA F/V, and FcgRIIIB NA1/NA2.

In bold: significant P value at the 0.05 threshold; underlined: trend of significance at the P value threshold of 0.05.

The analysis of the interaction between FcgRIIA/RIIIA/RIIIB combined genotypes and environmental exposure showed that infants carrying 131RH/176FV/NA1NA2 compared to 131RR/176FF/NA2NA2 have a trend towards a lower number of infections when exposed to low environmental risk levels (n = 13, IRR = 0.445, 95% CI = 0.194;1.017, P = 0.055). However, compared to the same reference, the carriage of 131HH/176FF/NA1NA1 was associated with a trend towards a higher risk of infection when exposed to low levels of exposure (n = 2, IRR = 2.622, 95% CI = 0.978;7.029, P = 0.055) (**Table 8**).

**Table 8:** Interaction between FcgRIIA/RIIIA/RIIIB combined genotypes—environmental exposure and malaria infections.

FcgR genotypes combinations*exposure to malaria	n	IRR	95% CI	P value
131RR/176FF/NA2NA2*low exposure (reference)	9			
131RR/176FF/NA2NA2*high exposure	12	0.737	0.356; 1.524	0.411
131RR/176FF/NA1NA2*low exposure	10	0.947	0.498; 1.801	0.869
131RR/176FF/NA1NA2*high exposure	16	0.729	0.354; 1.503	0.393
131RR/176FF/NA1NA1*low exposure	5	1.078	0.508; 2.284	0.844
131RR/176FF/NA1NA1*high exposure	4	1.293	0.573; 2.917	0.535
131RR/176FV/NA2NA2*low exposure	7	1.052	0.513; 2.158	0.889
131RR/176FV/NA2NA2*high exposure	5	1.230	0.486; 3.113	0.662
131RR/176FV/NA1NA2*low exposure	3	0.219	0.028; 1.692	0.146
131RR/176FV/NA1NA2*high exposure	7	0.488	0.185; 1.286	0.147
131RR/176FV/NA1NA1*low exposure	3	0.880	0.284; 2.726	0.825
131RR/176FV/NA1NA1*high exposure	1	0.621	0.138; 2.793	0.535
131RR/176VV/NA1NA2*low exposure	1	0.796	0.224; 2.821	0.724
131RH/176FF/NA2NA2*low exposure	14	1.018	0.536; 1.933	0.955
131RH/176FF/NA2NA2*high exposure	8	1.385	0.728; 2.634	0.320
131RH/176FF/NA1NA2*low exposure	11	0.801	0.403; 1.595	0.529
131RH/176FF/NA1NA2*high exposure	15	0.883	0.464; 1.680	0.706
131RH/176FF/NA1NA1*low exposure	10	0.998	0.478; 2.084	0.997
131RH/176FF/NA1NA1*high exposure	21	1.176	0.638; 2.169	0.602
131RH/176FV/NA2NA2*low exposure	23	0.952	0.504; 1.799	0.881
131RH/176FV/NA2NA2*high exposure	14	1.287	0.685; 2.418	0.431
131RH/176FV/NA1NA2*low exposure	13	0.445	0.194; 1.017	0.055
131RH/176FV/NA1NA2*high exposure	10	1.147	0.598; 2.201	0.679
131RH/176FV/NA1NA1*low exposure	17	0.752	0.381; 1.484	0.412
131RH/176FV/NA1NA1*high exposure	15	1.207	0.629; 2.318	0.571
131RH/176VV/NA2NA2*low exposure	5	0.642	0.206; 2.001	0.445
131RH/176VV/NA1NA2*low exposure	3	1.778	0.720; 4.386	0.211
131RH/176VV/NA1NA2*high exposure	3	1.675	0.761; 3.688	0.200
131RH/176VV/NA1NA1*low exposure	2	0.381	0.049; 2.933	0.355
131RH/176VV/NA1NA1*high exposure	3	0.733	0.164; 3.280	0.685
131HH/176FF/NA2NA2*low exposure	2	1.013	0.286; 3.591	0.983
131HH/176FF/NA2NA2*high exposure	3	1.855	0.746; 4.609	0.183
131HH/176FF/NA1NA2*low exposure	3	0.481	0.134; 1.719	0.260
131HH/176FF/NA1NA2*high exposure	3	0.454	0.058; 3.496	0.449
131HH/176FF/NA1NA1*low exposure	2	2.622	0.978; 7.029	0.055
131HH/176FF/NA1NA1*high exposure	5	1.332	0.498; 3.559	0.567
131HH/176FV/NA2NA2*high exposure	7	0.943	0.375; 2.371	0.901
131HH/176FV/NA1NA2*low exposure	10	1.033	0.504; 2.118	0.927
131HH/176FV/NA1NA2*high exposure	10	0.955	0.486; 1.876	0.895
131HH/176FV/NA1NA1*low exposure	6	0.362	0.103; 1.275	0.114
131HH/176FV/NA1NA1*high exposure	8	0.791	0.381; 1.641	0.530
131HH/176VV/NA2NA2*low exposure	2	1.520	0.535; 4.319	0.431
131HH/176VV/NA2NA2*high exposure	3	1.724	0.835; 3.559	0.140
131HH/176VV/NA1NA2*low exposure	4	0.366	0.047; 2.828	0.336
131HH/176VV/NA1NA2*high exposure	5	1.888	0.922; 3.867	0.082
131HH/176VV/NA1NA1*low exposure	2	1.328	0.430; 4.099	0.622
131HH/176VV/NA1NA1*high exposure	2	1.752	0.673; 4.562	0.251

This table presents the zero inflated negative binomial model, with the interaction between FcgR and environmental exposure obtained through the control variables mother age (in years), child age (in months), number of antenatal visits, and use of bednet.

Underlined: trend of significance at the P value threshold of 0.05.

### Detection of Gene-Gene and Gene-Environment Interactions by Generalized Multifactor Dimensionality Reduction (GMDR)

GMDR was used to screen the potential interactions among G3m and FcgR polymorphisms and to evaluate the impact of gene-exposure interaction on the risk of malaria infection.

Among all possible one- to four-locus models evaluated by the GMDR method, none reached the cut-off significance level of 0.05 when mosquito exposure was included as a covariate in the GMDR models, along with mother and child age, bednet use, and number of antenatal visits (data not shown). In contrast, when the exposure variable was combined with G3m and FcgR polymorphisms to detect potential gene-environment interactions, several models displayed significant results (**Table 9**).

**Table 9:** Association between FcgR polymorphisms, G3m phenotypes, and malaria infections: Results of the GMDR analysis when considering environmental exposure as an interaction variable.

No. of variables considered	Best model	Testing balanced accuracy(TBA)	Sign testP-value	Cross-validation consistency (CVC)
1	environmental exposure	0.6161	**0.0107**	10/10
2	FcgRIIA, environmental exposure	0.6038	**0.0010**	9/10
3	FcgRIIA, FcgRIIIA, environmental exposure	0.4871	0.6230	5/10
4	FcgRIIA, FcgRIIIA, FcgRIIIB, environmental exposure	0.6082	**0.0010**	10/10
5	G3m^†^, FcgRIIA, FcgRIIIA, FcgRIIIB, environmental exposure	0.6270	**0.0010**	10/10

All models are adjusted for mother age (in years), child age (in months), bednet use, and number of antenatal visits.

^†^These results were obtained only for the four following G3m allotypes and allotype combinations: G3m5,6,11,24, G3m5,6,10,11,13,15,24, G3m10, and G3m13 (each considered as presence/absence). The five-factor models were also significant for G3m5,10,11,13,14,15 (TBA = 0.5602; CVC = 10/10) and G3m14 (TBA = 0.6043; CVC = 10/10) although at a lesser degree (sign test P = 0.0107 in both cases).

In bold: significant P value at the 0.05 threshold.

The five-factor interaction model combining G3m, FcgRIIA, FcgRIIIA, FcgRIIIB polymorphisms, and mosquito exposure was the best model identified, with the maximum prediction accuracy of 62.70%, the maximum CV consistency of 10/10, and a sign test P-value of 0.001.

These results only hold for four G3m in single form and in combinations: G3m5,6,11,24, G3m5,6,10,11,13,15,24, G3m10, and G3m13. Models with lower prediction accuracies, though still significant, were obtained for G3m5,10,11,13,14,15 and G3m14 (TBA of 0.5602 and 0.6043, respectively; CVC = 10/10 and P = 0.011 for both). These results suggest significant gene-gene interactions between G3m and FcgR polymorphisms that are only revealed in an environment of high exposure to mosquito bites. The four-factor model including the three FcgR polymorphisms and mosquito exposure was the second best prediction model with a prediction accuracy of 60.82%, a maximum CV consistency of 10/10, and a sign test P-value of 0.001. The two-factor model involving the FcgRIIA 131R/H polymorphism and mosquito exposure (TBA of 0.604) reached an almost identical prediction accuracy.

We also conducted a linear regression analysis for the significant models associating G3m, FcgR and environmental exposure found in the GMDR analysis (**Table 9**, P in bold) in order to estimate the effect of such associations. GMDR analysis is based on linear regression. Therefore, using linear regression models was perfectly suited to quantify the effect detected by GMDR analysis. Consistent with the GMDR analysis, the linear regression models showed a higher risk of malaria infection in carriers of 131RR/176FF/NA2NA2/G3m5,6,11,24 and 131HH/176VV/NA1NA2/G3m5,6,10,11,13,15,24 combinations, when exposed to a high-risk environment (regression coefficient = 4.804, P = 0.032 and regression coefficient = 5.444, P = 0.015, respectively) (**Supplementary Table 1**). Linear models confirmed, also, a higher risk of malaria infection in carriers of G3m10 and G3m13 combined to 131HH/176FF/NA1NA1 in a low-risk environment with a regression coefficient of 6.160 (P = 0.039) and 6.248 (P = 0.044), respectively (**Supplementary Table 2**). The genotype combination 131HH/176FF/NA1NA1 indeed showed a trend towards a higher risk of infection in a low-risk environment (**Table 8**).

## Discussion

**Figure 3:**
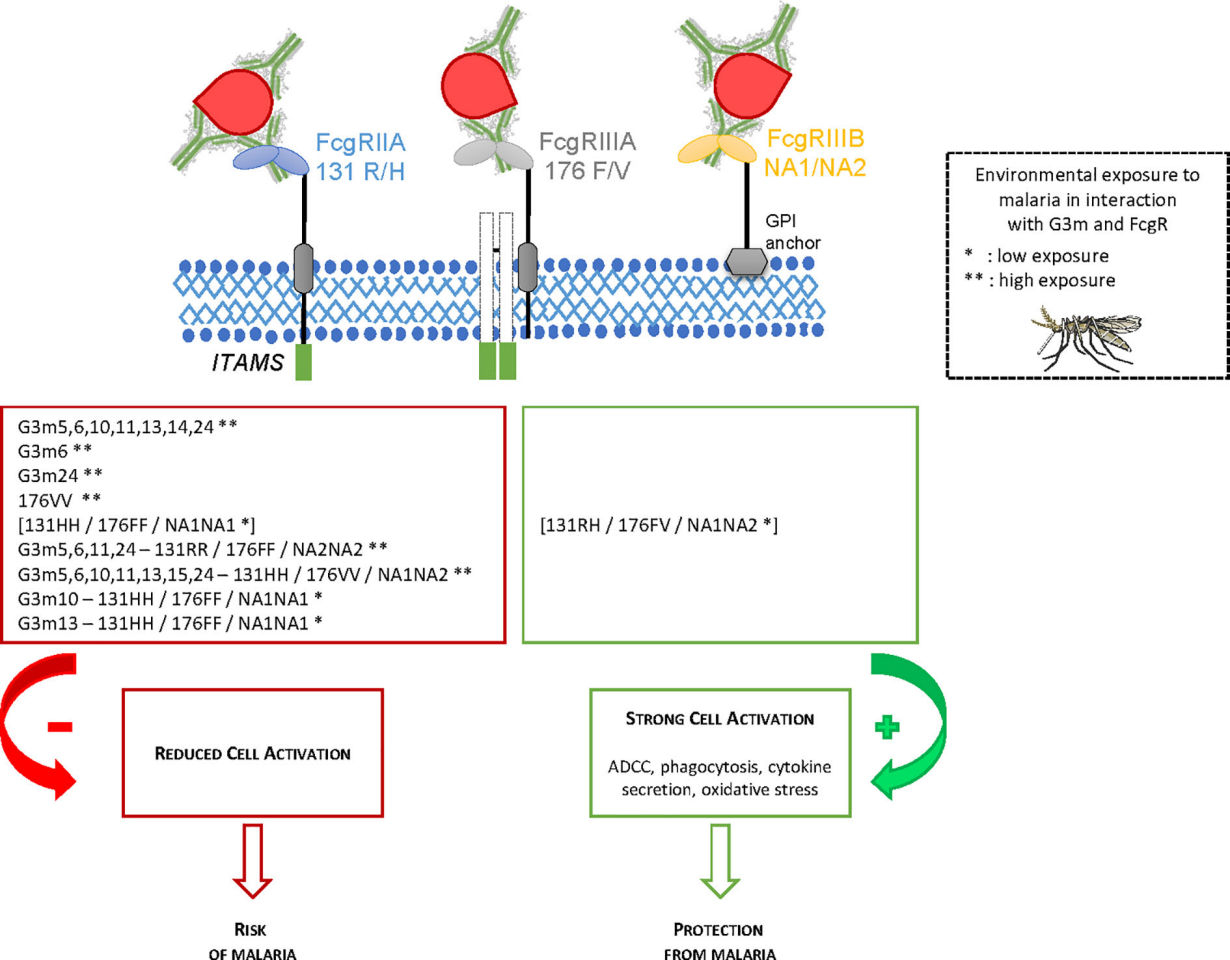
Summary of the main findings of the study.

We studied the combined impact of FcgRIIA, FcgRIIIA, and FcgRIIIB polymorphisms and IgG G3m allotypes on malaria susceptibility in early life in Benin. First, we studied separately the influence of IgG G3m allotypes and of FcgRs polymorphisms on the number of *P. falciparum* infections during an 18 months clinical and parasitological follow-up of newborns. Second, the influence of combined IgG G3m allotypes and FcgRs polymorphisms was assessed. We identified an increased risk of *P. falciparum* infection in infants carrying particular FcgRIIA 131R/H – FcgRIIIA 176F/V – FcgRIIIB NA1/NA2 genotypes combined to G3m5,6,11,24 or G3m5,6,10,11,13,15,24 phenotypes or to G3m10 or G3m13 single allotypes. These observations were reinforced by the application of two complementary statistical approaches (GMDR method and linear regression analysis), allowing to take into account the individual risk of exposure to malaria. **Figure 3** presents a summary of the main results.

No diversity was observed among infants for the G1m allotypes, since all infants were G1m1 and G1m17 positive; therefore, the analysis focused on the G3m diversity present on IgG3. Our results showed an increased risk of malaria infections associated with the carriage of the G3m5,6,10,11,13,14,24 phenotype. It should be pointed out that the presence of G3m24 in this combination could play an important role as it was also associated with a higher risk to be infected by *P. falciparum* when considered as a single allotype. The risk effect of some allotypes was accentuated when infants were highly exposed to mosquito bites. For example, the effect of G3m6 appeared only when the interaction with the exposure variable was considered. These results suggest that the G3m6 effect is accentuated in conditions of high mosquito exposure and the risk associated with G3m5,6,10,11,13,14,24 could be linked to the presence of both G3m6 and G3m24.

The interactions observed between G3m allotypes and mosquito exposure could be related to the quantity of anti-malarial IgG3 that increased following repeated infections with *P. falciparum*. IgG3 antibodies were shown to be protective against malaria and they can act directly by limiting host cell invasion or indirectly *via* their Fc-mediated eﬀector functions (8, 42). Therefore, an increased level of IgG3 harboring specific G3m allotypes, in case of high mosquito exposure, could facilitate the identification of the risk effect linked to these characteristics. Of note, IgG allotypes were found to correlate with serum IgG levels (19, 43) but also with the switching between IgG isotypes suggesting that IgG allotypes may aﬀect the humoral antibody response. Moreover, it was recently demonstrated that allotypic variants within the IgG3 subclass substantially aﬀect FcgRIIIA binding and Antibody-Dependent Cellular Cytotoxicity (44). To our knowledge, no work has previously shown associations between Gm phenotypes comprising G3m24 or G3m24 alone with a higher risk of malaria infections, while G3m24 is prevalent among sub-Saharan African populations (45, 46). In contrast, Gm phenotypes including G3m6 have been already associated with malaria susceptibility (10, 17, 18). Indeed, the G3m5,6,13,14 phenotype was associated with a higher incidence of uncomplicated malaria (18). It is interesting to note that G3m6 allotype is rare among Fulani, who are less susceptible to malaria than other sympatric ethnic groups (17). However, we also demonstrated the existence of an inverse relationship between the carriage of the G3m5,6,13,14 phenotype and the presence of uncomplicated malaria (10) in the Fon ethnic group. Given that both polymorphisms in the IgG3 heavy chain gene and FcgR can influence the IgG binding on immunoglobulin Fc-receptors and impact immunity to malaria through ADCC, ADCI, or phagocytosis of malaria parasites, the interpretation of results coming from separate investigations of these distinct polymorphisms remains challenging.

FcgRs provide a crucial link between the humoral and cellular immune responses. Polymorphisms which alter the affinity of FcgRs in binding IgG subclasses have been described. In human, FcgRII and FcgRIII are known to bind IgG subclasses. Regarding FcgRIIA 131R/H polymorphisms, our results revealed no significant association with malaria infections whether or not the mosquito exposure factor was taken into account. On the other side, a malaria risk associated with the FcgRIIIA 176VV genotype was observed, which was strengthened in conditions of high exposure to mosquitoes. These results differed from those of Omi et al. (47) who found no relation between FcgRIIIA 176F/V polymorphism and the severity of malaria in Thai people (47) and of Munde et al. (11) who showed no relation between FcgRIIIA 176F/V and severe malarial anemia (SMA) (11). The differences in the size of the population groups, the ethnic origin and genetic diversity of the populations and the clinical definition of malaria used, may preclude a rigorous comparison between studies. In other cases, the FcgRIIIA 176V allele was identified as a genetic risk factor for the development of atopic diseases (48) or for generalized aggressive periodontitis (39). It has been shown that the 176V variant improved the FcgRIIIA affinity for IgG1 and IgG2 (49). Regarding our results, this same variant could be associated with a lower affinity to IgG3, thus reducing the effectiveness of the antimalarial response. Finally, our results showed an association between the FcgRIIIB NA1NA2 genotype and protection against malaria when compared to the carriage of NA2NA2, while in conditions of high mosquito exposure, the NA2NA2 genotype was associated with a higher risk compared to NA1NA1. The polymorphic expression of FcgRIIIB NA1/NA2 influences the phagocytic capacity of neutrophils and Salmon et al. (50) already showed a decreased phagocytosis in relation to NA2NA2 compared to NA1NA1 (50). In line with these results, other studies showed a risk for malaria (30) or cerebral malaria (29) associated with the carriage of the FcgRIIIB NA2 variant.

FcgR receptors act synergistically by crosslinking. The additive effects of the host FcgRIIA/FcgRIIIA/FcgRIIIB genotypes might impact the immune response to *P. falciparum* and therefore affect the outcome of the disease. This synergy function results in phagocytosis of immunoglobulin-opsonized immune complexes and in the stimulation of neutrophils degranulation, which leads to the production of reactive oxygen species (ROS). The absence in our study of relationship with malaria infection involving FcgRIIA/FcgRIIIA genotype combinations compared to FcgRIIA/FcgRIIIA/FcgRIIIB ones may reflect a particular synergy between FcgRIIA and FcgRIIIB receptors (**Table 9**). Indeed, it has been shown that FcgRIIA and FcgRIIIB can interact functionally to trigger neutrophils by means of an IgG mediated response (51–53). Moreover, FcgRIIA is considered essential for the induction of effector functions, and the abundance of FcgRIIIB can guarantee an effective interaction with IgG complexes (54). As already suggested, the HWE deviation observed for FcgRIIIB could be due to unidentified mutations likely resulting from disease-related evolutionary selection pressure exerted by *P. falciparum* and potentially by other infectious diseases occurring in the population (11).

It has been shown that FcgRIIA 131H contributes to an efficient binding to IgG2 and IgG3 as opposed to 131R (23) and also that IgG2 and IgG3 contribute to individual resistance to malaria (8). The 131H variant was found more prevalent in the Fulani people of Daraweesh, a village in eastern Sudan, who are less affected by clinical malaria (55). The same variant was associated with protection from malaria infection in Indian individuals at least 5 years old (56) and with protection against high parasitemia both in African and Asian people (31). However it was associated with a higher risk in a cohort of Gambian children less than 5 years (57). In comparison, the 131R variant has been shown to play a major role in ADRB (58, 59) and is associated with a low phagocytic activity and poor immune complex clearance (60). In genotype analysis, the higher risk associated with 131RH/176VV/NA1NA2, 131HH/176VV/NA2NA2, 131HH/176VV/NA1NA2 and 131HH/176FF/NA1NA1 carriage compared to 131RR/176FF/NA2NA2 may be explained by the presence of FcgRIIIA 176VV in the three first genotype combinations. Moreover, the joint presence of FcgRIIA 131H variant in all combinations is in line with results from some of the studies reported above, according to which this variant is less protective than the 131R one (57).

Since G3m and FcgR polymorphisms act individually on malaria infections, it is plausible that their combined polymorphisms act in synergy against malaria infections. This aspect has never been studied before and the MDR method was used to precisely explore it. The G3m5,6,11,24 – 131RR/176FF/NA2NA2 and G3m5,6,10,11,13,15,24 – 131HH/176VV/NA1NA2 combinations were the most significant G3m/FcgR ones associated with malaria (at risk according to the linear regressions) when taking into account the interaction with the environmental variable of exposure to mosquito bites, according to the GMDR analysis. This is particularly interesting in view of the preceding results, indicative of a cumulative risk linked to the double carriage of G3m6 and G3m24 joined to either FcgRIIIA 176VV or FcgRIIIB NA2NA2. Moreover, linear regression of these interactions showed high regression coefficients (4.804 and 5.444). Thus, these interactions are at risk under conditions of high exposure to mosquito bites because of the cumulative presence of G3m6 and G3m24 in one hand and of the FcgRIIIA 176VV and FcgRIIIB NA2NA2 genotypes in another hand. The malaria risk under conditions of low exposure involving G3m10 - FcgRIIA 131HH/FcgRIIIA 176FF/FcgRIIIB NA1NA1 and G3m13 - FcgRIIA 131HH/FcgRIIIA 176FF/FcgRIIIB NA1NA1 is interesting to note since at the individual level, G3m10 and G3m13 did not show any significant association. Thus, this result could reflect the fact that the effect found is only due to the influence of the FcgR association. Moreover, the linear regression showed that the FcgR associations concerned were defined at risk in the previous results.

The efficacy of IgG3 response can be explained by FcgR affinity but also by the nature of targeted antigens. High concentrations of AMA1, MSP1, MSP2-FC27, MSP3, GLURP-R2-specific IgG3 were found in infants able to control asymptomatic infections (61). Of course, other key antigens expressed during the different steps of the *P. falciparum* cycle life probably participate to the control of the parasite growth. Functional assays will clarify the mechanisms and antigens involved in malaria protection. These answers can probably also vary depending immune system cells. For example, FcgRIIIB is present on neutrophils and not monocytes, natural killers react more with FcgRIIIA compared to FcgRIIA, while monocytes and macrophages present both FcgRIIA and FcgRIIIA.

This study showed the importance of polymorphisms in both FcgR and IgG in the modulation of the risk of malaria infections in Beninese infants. It would then be interesting to look at the CNV (Copy Number Variations) of these polymorphisms and their influence on risk or protection against malaria. CNV are polymorphisms represented by DNA segments that differ among individuals due to suppression, insertion, inversion, duplication, or complex recombination (62). Recent studies have highlighted the relationship between CNV and disease: FcgRIIIB low CNV was associated with systemic lupus erythematosus risk (63) while for Chen et al. (64) a low FcgRIIIA CNV was positively associated with lupus and rheumatoid arthritis and a low FcgRIIIB CNV, with a risk of lupus but not rheumatoid arthritis (64). It would be interesting, thus, to explore the role of CNV in FcgR receptors in individual variation of malaria susceptibility.

In summary, the current study demonstrates that some combined G3m-FcgR polymorphisms are associated with a malaria risk and that this risk is even more pronounced in case of high mosquito exposure. The results highlight the relevance of studying combined IgG heavy chain/FcgR polymorphisms in relation to *P. falciparum* malaria as one or the other, or both, may influence the individual susceptibility to infection. Understanding the functional diversity within IgG subclasses may shed light on associations found with infectious diseases or auto-immune diseases and potentially initiate new strategies to improve therapeutic antibodies.

## Data Availability Statement

The data presented in the study are deposited in the Open Science Framework repository (https://osf.io/), with accession number gmr4s.

## Ethics Statement

The studies involving human participants were reviewed and approved by the Abomey-Calavi’s institutional review board and the IRD’s Consultative Ethics Committee. Written informed consent to participate in this study was provided by the participants’ legal guardian/next of kin.

## Author Contributions

CD, DC, and FM-N conceived and designed the project. AF, EG, DC, and FM-N performed the laboratory experiments. JM and J-MD brought technical support or result validation. AF, CD, AS, AG, DC, and FM-N performed statistical analysis. AF, CD, DC, and FM-N wrote the paper. All authors contributed to the article and approved the submitted version.

## Funding

This work was supported by the Agence Nationale de la Recherche (ANR) Santé Environnement Santé Travail (SEST 2006; 040 01). The Université de Paris awarded a PhD scholarship to AF.

## Conflict of Interest

The authors declare that the research was conducted in the absence of any commercial or financial relationships that could be construed as a potential conflict of interest.
