# Supplementary material for: Susceptibility to Plasmodium falciparum Malaria: Influence of Combined Polymorphisms of IgG3 Gm Allotypes and Fc Gamma Receptors IIA, IIIA, and IIIB
Source: Front Immunol. 2020 Dec 23;11:608016. doi: 10.3389/fimmu.2020.608016 (PMC7786284; doi:10.3389/fimmu.2020.608016)
Supplement: Supplementary file 1 [file Table_1.docx]

**Supplementary data: Linear regressions for GMDR significant models**

**Supplementary Table 1: Linear regression for significant G3m phenotypes**

| Linear regression of G3m5,6,11,24, FcgR and environmental exposure | | | |
| --- | --- | --- | --- |
|  | **Coef.** | **P value** | **95% CI** |
| G3m5,6,11,24 - 131RR/176FF/NA2NA2*low exposure | -0.981 | 0.660 | -5.369 ; 3.406 |
| G3m5,6,11,24 - 131RR/176FF/NA2NA2*high exposure | **4.804** | **0.032** | **0.408 ; 9.199** |
| G3m5,6,11,24 - 131RR/176FF/NA1NA2*low exposure | 1.292 | 0.563 | -3.100 ; 5.686 |
| G3m5,6,11,24 - 131RR/176FF/NA1NA2*high exposure | -0.131 | 0.953 | -4.523 ; 4.261 |
| G3m5,6,11,24 - 131RR/176FF/NA1NA1*low exposure | 0.035 | 0.987 | -4.447 ; 4.519 |
| G3m5,6,11,24 - 131RR/176FF/NA1NA1*high exposure | 1.592 | 0.482 | -2.857 ; 6.042 |
| G3m5,6,11,24 - 131RH/176FF/NA2NA2*low exposure | -0.293 | 0.895 | -4.693 ; 4.105 |
| G3m5,6,11,24 - 131RH/176FF/NA1NA2*high exposure | 0.765 | 0.732 | -3.635 ; 5.167 |
| G3m5,6,11,24 - 131RH/176FF/NA1NA1*low exposure | -0.955 | 0.966 | -4.508 ; 4.317 |
| G3m5,6,11,24 - 131RH/176FF/NA1NA1*high exposure | -2.620 | 0.243 | -7.027 ; 1.786 |
| G3m5,6,11,24 - 131RH/176FV/NA2NA2*low exposure | -3.280 | 0.146 | -7.711 ; 1.149 |
| G3m5,6,11,24 - 131RH/176FV/NA1NA2*high exposure | -0.373 | 0.867 | -4.778 ; 4.030 |
| G3m5,6,11,24 - 131RH/176FV/NA1NA1*low exposure | 0.879 | 0.603 | -2.443 ; 4.202 |
| G3m5,6,11,24 - 131RH/176FV/NA1NA1*high exposure | 0.132 | 0.953 | -4.290 ; 4.555 |
| G3m5,6,11,24 - 131HH/176FV/NA2NA2*high exposure | -2.208 | 0.123 | -5.021 ; 0.604 |
| G3m5,6,11,24 - 131HH/176FV/NA1NA2*low exposure | 2.152 | 0.335 | -2.240 ; 6.545 |
| G3m5,6,11,24 - 131HH/176FV/NA1NA2*high exposure | 1.149 | 0.490 | -2.122 ; 4.420 |
| G3m5,6,11,24 - 131HH/176VV/NA2NA2*high exposure | 1.564 | 0.349 | -1.722 ; 4.850 |
|  | | | |
| Linear regression of G3m5,6,10,11,13,15,24, FcgR and environmental exposure | | | |
|  | **Coef.** | **P value** | **95% CI** |
| G3m5,6,10,11,13,15,24 - 131RR/176FF/NA1NA2*low exposure | -0.540 | 0.809 | -4.925 ; 3.844 |
| G3m5,6,10,11,13,15,24 - 131RR/176FV/NA2NA2*low exposure | 0.052 | 0.982 | -4.429 ; 4.535 |
| G3m5,6,10,11,13,15,24 - 131RH/176FF/NA2NA2*high exposure | 2.643 | 0.237 | -1.746 ; 7.034 |
| G3m5,6,10,11,13,15,24 - 131RH/176FF/NA1NA2*low exposure | -3.743 | 0.098 | -8.179 ; 0.692 |
| G3m5,6,10,11,13,15,24 - 131RH/176FF/NA1NA1*high exposure | -0.765 | 0.735 | -5.225 ; 3.693 |
| G3m5,6,10,11,13,15,24 - 131RH/176FV/NA2NA2*low exposure | 0.750 | 0.737 | -3.647 ; 5.148 |
| G3m5,6,10,11,13,15,24 - 131RH/176FV/NA2NA2*high exposure | -0.664 | 0.688 | -3.924 ; 2.594 |
| G3m5,6,10,11,13,15,24 - 131RH/176FV/NA1NA2*high exposure | 1.470 | 0.513 | -2.948 ; 5.889 |
| G3m5,6,10,11,13,15,24 - 131RH/176FV/NA1NA1*low exposure | -0.297 | 0.894 | -4.713 ; 4.118 |
| G3m5,6,10,11,13,15,24 - 131HH/176FV/NA1NA2*low exposure | -0.648 | 0.773 | -5.073 ; 3.777 |
| G3m5,6,10,11,13,15,24 - 131HH/176FV/NA1NA1*high exposure | -1.402 | 0.533 | -5.825 ; 3.021 |
| G3m5,6,10,11,13,15,24 - 131HH/176VV/NA1NA2*high exposure | **5.444** | **0.015** | **1.056 ; 9.831** |
| G3m5,6,10,11,13,15,24 - 131HH/176VV/NA1NA1*low exposure | 0.407 | 0.805 | -2.842 ; 3.657 |

**Supplementary Table 2: Linear regression for significant G3m single allotypes**

| Linear regression of G3m10, FcgR and environmental exposure | | | |
| --- | --- | --- | --- |
|  | **Coef.** | **P value** | **95% CI** |
| G3m10 - 131RR/176FF/NA2NA2*low exposure | 0.981 | 0.660 | -3.406 ; 5.369 |
| G3m10 - 131RR/176FF/NA1NA2*high exposure | 0.274 | 0.902 | -4.120 ; 4.670 |
| G3m10 - 131RR/176FF/NA1NA2*low exposure | 1.389 | 0.524 | -2.89 ; 5.674 |
| G3m10 - 131RR/176FF/NA2NA2*high exposure | 0.959 | 0.660 | 3.334 ; 5.253 |
| G3m10 - 131RR/176FF/NA1NA1*low exposure | 1.700 | 0.484 | -3.078 ; 6.479 |
| G3m10 - 131RR/176FF/NA1NA1*high exposure | 3.599 | 0.165 | -1.487 ; 8.686 |
| G3m10 - 131RR/176FV/NA2NA2*low exposure | 1.867 | 0.406 | -2.555 ; 6.291 |
| G3m10 - 131RR/176FV/NA2NA2*high exposure | 0.591 | 0.798 | -3.943 ; 5.125 |
| G3m10 - 131RR/176FV/NA1NA2*low exposure | -1.125 | 0.708 | -7.036 ; 4.785 |
| G3m10 - 131RR/176FV/NA1NA2*high exposure | 0.170 | 0.940 | -4.257 ; 4.598 |
| G3m10 - 131RR/176FV/NA1NA1*low exposure | 0.623 | 0.797 | -4.150 ; 5.398 |
| G3m10 - 131RR/176FV/NA1NA1*high exposure | 1.301 | 0.664 | -4.590 ; 7.194 |
| G3m10 - 131RR/176VV/NA1NA2*low exposure | 1.275 | 0.669 | -4.592 ; 7.143 |
| G3m10 - 131RH/176FF/NA2NA2*low exposure | 1.79 | 0.413 | -2.505 ; 6.085 |
| G3m10 - 131RH/176FF/NA2NA2*high exposure | 3.305 | 0.140 | -1.088 ; 7.700 |
| G3m10 - 131RH/176FF/NA1NA2*low exposure | 0.537 | 0.806 | -3.773 ; 4.849 |
| G3m10 - 131RH/176FF/NA1NA2*high exposure | 1.249 | 0.570 | -3.079 ; 5.578 |
| G3m10 - 131RH/176FF/NA1NA1*low exposure | 1.011 | 0.648 | -3.344 ; 5.367 |
| G3m10 - 131RH/176FF/NA1NA1*high exposure | 2.068 | 0.340 | -2.188 ; 6.324 |
| G3m10 - 131RH/176FV/NA2NA2*low exposure | 0.863 | 0.689 | -3.381 ; 5.108 |
| G3m10 - 131RH/176FV/NA2NA2*high exposure | 2.148 | 0.326 | -2.148 ; 6.445 |
| G3m10 - 131RH/176FV/NA1NA2*low exposure | -0.822 | 0.970 | -4.377 ; 4.212 |
| G3m10 - 131RH/176FV/NA1NA2*high exposure | 2.365 | 0.291 | -2.035 ; 6.767 |
| G3m10 - 131RH/176FV/NA1NA1*low exposure | 0.579 | 0.790 | -3.701 ; 4.859 |
| G3m10 - 131RH/176FV/NA1NA1*high exposure | 1.772 | 0.417 | -2.517 ; 6.063 |
| G3m10 - 131RH/176VV/NA2NA2*low exposure | 0.251 | 0.913 | -4.280 ; 4.783 |
| G3m10 - 131RH/176VV/NA2NA2*high exposure | -1.617 | 0.588 | -7.498 ; 4.262 |
| G3m10 - 131RH/176VV/NA1NA2*low exposure | 4.118 | 0.111 | -0.951 ; 9.189 |
| G3m10 - 131RH/176VV/NA1NA2*high exposure | 4.578 | 0.077 | -0.501 ; 9.658 |
| G3m10 - 131RH/176VV/NA1NA1*low exposure | -0.536 | 0.835 | -5.606 ; 4.533 |
| G3m10 - 131RH/176VV/NA1NA1*high exposure | 0.313 | 0.903 | -4.768 ; 5.394 |
| G3m10 - 131HH/176FF/NA2NA2*low exposure | 0.369 | 0.887 | -4.730 ; 5.469 |
| G3m10 - 131HH/176FF/NA2NA2*high exposure | 4.087 | 0.116 | -1.018 ; 9.194 |
| G3m10 - 131HH/176FF/NA1NA2*low exposure | 0.114 | 0.962 | -4.673 ; 4.902 |
| G3m10 - 131HH/176FF/NA1NA2*high exposure | 0.551 | 0.830 | -4.507 ; 5.610 |
| G3m10 - 131HH/176FF/NA1NA1*low exposure | **6.160** | **0.039** | **0.306 ; 12.014** |
| G3m10 - 131HH/176FF/NA1NA1*high exposure | 1.665 | 0.492 | -3.104 ; 6.435 |
| G3m10 - 131HH/176FV/NA2NA2*low exposure | -1.131 | 0.661 | -6.203 ; 3.941 |
| G3m10 - 131HH/176FV/NA2NA2*high exposure | 1.850 | 0.473 | -3.221 ; 6.922 |
| G3m10 - 131HH/176FV/NA1NA2*low exposure | 0.809 | 0.714 | -3.532 ; 5.151 |
| G3m10 - 131HH/176FV/NA1NA2*high exposure | 1.814 | 0.421 | -2.616 ; 6.245 |
| G3m10 - 131HH/176FV/NA1NA1*low exposure | 0.144 | 0.951 | -4.483 ; 4.772 |
| G3m10 - 131HH/176FV/NA1NA1*high exposure | 1.062 | 0.634 | -3.329 ; 5.454 |
| G3m10 - 131HH/176VV/NA2NA2*low exposure | 3.470 | 0.245 | -2.391 ; 9.331 |
| G3m10 - 131HH/176VV/NA2NA2*high exposure | 4.156 | 0.110 | -0.945 ; 9.257 |
| G3m10 - 131HH/176VV/NA1NA2*low exposure | 0.703 | 0.772 | -4.080 ; 5.488 |
| G3m10 - 131HH/176VV/NA1NA2*high exposure | 3.131 | 0.176 | -1.410 ; 7.673 |
| G3m10 - 131HH/176VV/NA1NA1*low exposure | 1.271 | 0.621 | -3.792 ; 6.335 |
| G3m10 - 131HH/176VV/NA1NA1*high exposure | 2.448 | 0.343 | -2.622 ; 7.518 |
|  | | | |
| Linear regression of G3m13,FcgR and environmental exposure | | | |
|  | **Coef.** | **P value** | **95% CI** |
| G3m13 - 131HH/176FF/NA1NA1*low exposure 6.248 0.044 0.165 ; 12.332 | | | |
| G3m13 - 131RH/176VV/NA1NA2*high exposure 4.545 0.091 -0.733 ; 9.824 | | | |

**Linear regression for trendy significant G3m single allotypes**

| Linear regression of G3m14, FcgR and environmental exposure | | | |
| --- | --- | --- | --- |
|  | **Coef.** | **P value** | **95% CI** |
| G3m14 - 131HH/176FF/NA2NA2*low exposure 6.294 0.041 0.254 ; 12.335 | | | |
| G3m14 - 131RH/176VV/NA1NA2*high exposure 4.638 0.083 -0.601 ; 9.879 | | | |
